# CER818: A Highly Specific and Sensitive HPV L1 High-Risk Serological Lateral Flow Rapid Test for Early Detection of Cervical Cancer and Its Precursor Lesions

**DOI:** 10.1155/2024/6651272

**Published:** 2024-07-30

**Authors:** Karen Bräutigam, Stefanie Meier, Frank Köster, Achim Rody, Ralf Hilfrich

**Affiliations:** ^1^ Department of Gynecology and Obstetrics University of Lübeck and University Medical Center Schleswig-Holstein, Campus Lübeck, Ratzeburger Allee 160 23560, Lübeck, Germany; ^2^ Section for Translational Surgical Oncology and Biobanking Department of Surgery University of Lübeck and University Medical Center Schleswig-Holstein, Campus Lübeck, Ratzeburger Allee 160 23560, Lübeck, Germany; ^3^ Abcerion Diagnostics GmbH R&D, Zum Roemberg 24 65597, Huenfelden, Germany

**Keywords:** cervical cancer screening, CIN, HPV high risk, L1 serology, LMICs

## Abstract

**Objective:** The objective of the study is to validate a new human papillomavirus (HPV) L1 high-risk specific serological assay in a case-control study.

**Methods:** Serum samples of 138 patients (cervical intraepithelial neoplasia (CIN) 1, 2, and 3 and cervical cancer), 21 vaccinees, and 246 female controls were tested for the presence of HPV L1 high-risk specific antibodies.

**Results:** HPV L1 high-risk antibodies were detected in 100% of the CIN1 and 2, 86.6% of the CIN3 and 82.4% of the cervical cancer cases, 100% of the vaccinees, and 3.9% of the female controls. Area under the curve (AUC) was calculated with 0.91 for controls versus CIN2+, 0.923 for controls versus CIN1+, and 0.968 for controls versus CIN1/2.

**Conclusion:** The HPV L1 high-risk specific serological lateral flow rapid test shows promising data in the field of early detection of HPV high-risk induced cervical cancer and its precursor lesions. This easy-to-use, robust, and affordable approach could offer a chance to reach women in low- or middle-income countries (LMICs) that could not be reached by HPV molecular testing–based cervical cancer screening programs.

## 1. Introduction

Cervical cancer is still a leading cause of cancer-related death in women worldwide. To change this, the Cervical Cancer Elimination Modelling Consortium (CCEMC) of the World Health Organization (WHO) has defined the goal to eradicate the disease during the next century and shortly to achieve a global coverage of 90% vaccination, 70% screening, and 90% treatment by 2030 [1].

In many countries, primary and secondary preventive measures such as human papillomavirus (HPV) vaccination and smear-based early detection systems, enabling Pap-based microscopic and HPV-based molecular biological examinations, have led to a significant reduction in mortality, if combined with effective treatment modalities.

Nevertheless, every year, 604,000 new cervical cancer cases are recorded, and about 342,000 women die of the disease [2]. Women in low- or middle-income countries (LMICs) still suffer most from the disease since primary and secondary preventive measures are for multiple reasons not yet available to a desired extent. These inequalities in access to screening tools result in ethnic, racial, and social disparities in the incidence and mortality of cervical cancer [3, 4]. On the other hand, even if cervical cancer screening programs are established, some sociocultural factors prevent women from undergoing gynecological inspection, which results in a higher risk of mortality from cervical cancer in certain population groups [4].

Easy-to-use, non–smear-based HPV detection options may therefore be of advantage to change this and to enable each and every woman to become aware of her HPV-induced cervical cancer status.

As a “non–smear”-based alternative, urine sampling for HPV high-risk detection has shown to offer a comfortable method of sample collection [5–8]. The pooled sensitivity of urinary HPV test is with 84% significantly lower than that of clinician-collected samples, but still remarkably high, so that it seems to be a decent alternative screening tool [9]. Nevertheless, a centralized institute for HPV determination and logistic effort is necessary to establish such a screening program. Especially in rural areas, where no postal address is available to report the test result, it might emerge the situation that the test result does not reach the positively tested women. Near-patient care diagnosis could be of critical importance to change this situation to be able to combine screening and immediate treatment in a “see and treat approach” in LMICs.

Blood-based test systems are very often the standard of care for early diagnosis of virological diseases, but not for HPV. In the past, the development of such assays was hampered by the fact that HPV infection does not necessarily lead to a functional disorder, and vice versa, the presence of traditionally measured HPV antibodies was no proof for HPV-driven disease [10–12]. HPV serology was therefore thought to be a marker of infection but not disease. In 2020, Weiland et al. described for the first time a new blood-based HPV16-specific tumor marker, developed by one of us (RH), showing promising data by discriminating HPV-driven disease from subclinical HPV infection [13].

Due to the HPV16 specificity of this assay, it was obvious that such a test could only be of benefit for cervical cancer screening purposes by integrating additional HPV high-risk subtypes. Today, we report first results of an easy-to-use HPV high-risk specific serological rapid test, which may have the potential to be used in a “see and treat approach” especially in rural areas.

## 2. Methods

### 2.1. Study Population

For the analysis of the serological HPV L1 high-risk status, which was approved by the ethics committee of the University Medical Center Lübeck (AZ 17-155), in total, 405 (138 + 21 + 246) serum samples were collected between March 2019 and April 2022. One hundred thirty-eight cases (age 21–77, mean 41.5) with histologically confirmed CIN1, 2, and 3 or cervical cancer and 21 sera of vaccinated women (age 18–42, mean 27.8) visiting the outpatient unit were recruited at the Department of Gynecology and Obstetrics of the University Medical Center Schleswig-Holstein, Campus Lübeck, Germany. Histological diagnosis was carried out by two gynecological pathologists, who were not blinded to the original diagnosis. Blood samples were taken after the diagnosis and before treatment and were stored as serum at −20°C until analysis. A detailed description with additional patient information is given in Table 1 and has been reported previously [14].

As controls and to assess the diagnostic specificity of a healthy control group, 246 randomly selected serum samples of women (age 18–99, mean 45.8) were kindly provided by Laboratory Dr. Riegel (Wiesbaden, Germany). The 246 controls were selected out of six age decades with 40–44 controls each (see Table 1).

### 2.2. HPV Subtype Analysis of Cases

HPV sub–type-specific determination of the cases was carried out using the EUROArray HPV test (EUROIMMUN Medizinische Labordiagnostika AG, Lübeck, Germany). The test is based on amplification and detection of the viral oncogenes E6/E7 via PCR and hybridization with immobilized DNA probes in a microarray system. By using subtype-specific primer and probe systems, 30 anogenital HPV genotypes are detected and differentiated simultaneously in one multiplexed reaction: 18 HPV high risk (16, 18, 26, 31, 33, 35, 39, 45, 51, 52, 53, 56, 58, 59, 66, 68, 73, and 82) and 12 HPV low risk (6, 11, 40, 42, 43, 44, 54, 61, 70, 72, 81, and 89). The hybridization of the amplified product to the corresponding probe is detected using the EUROArrayScanner. The EUROArrayScan software subsequently evaluates all spot signals (relative fluorescent intensity) and generates a qualitative test result (i.e., detected/not detected based on HPV sub–type-specific cutoffs).

### 2.3. Competitive Serological Detection of Human Antibodies to HPV High-Risk L1 CER818 Epitope

Serological detection of HPV high-risk (HPV 16, 18, 31, 33, 35, 39, 45, 51, 52, 53, 56, 58, 68, and 73) L1-specific antibodies was carried out using a competitive CER818 epitope-specific rapid test (CancerCheck HPV High Risk, Concile GmbH, Freiburg, Germany), according to the manufacturer's instruction. In short, 25 *μ*L serum was mixed with an HPV high-risk specific reagent and transferred onto a lateral flow test cassette. After 15 min, the test result was measured using an Alpha 1 and an Omega 100 reader (both Concile GmbH, Freiburg, Germany). The purified mouse monoclonal antibody CER818 served as a standard for quantification.

### 2.4. Statistics

For demographic data, mean in numerical and percentages was calculated. Frequencies of positive test results were determined in number and percentages and used for sensitivity and specificity calculation. Comparison of CER818 (nanograms per milliliter) between controls and patients with intraepithelial neoplasia and cervical cancer was performed with multiple logistic regression and ROC (receiver operator characteristic) curve analyses with determination of the AUC (area under the curve). High statistical significance is given by *p* < 0.0001. All analyses were done by GraphPad Prism 6 for Windows (6.03).

## 3. Results

This retrospective analysis of HPV L1 high-risk specific CER818 antibodies included serum samples from 138 women with CIN1, 2, and 3 and cervical cancer, 21 vaccinated, and 246 randomly selected women as controls.

Seropositivity for HPV L1 high-risk CER818 was detected in pretreatment sera from 124 out of the 138 patients (89.9%) using a cutoff value of 750 ng/mL CER818 as shown in Table 2. All 39 CIN1 and CIN2 cases showed antibody levels above this value (sensitivity 100%). Seventy-one out of 82 CIN3 (86.6%) and 14 out 17 cervical cancer cases (82.4%) could be identified as well. Twenty-three cases (3 CIN1, 4 CIN2, and 16 CIN3) showed antibody levels between 750 and 1000 ng/mL, which results in an overall sensitivity choosing a cutoff value of 1000 ng/mL of still 72.5%.

Twenty out of 21 vaccinated women (95.2%) showed antibody levels above 1000 ng/mL.

The 246 women of the control group were chosen to six equally distributed age decades with 40–44 sera each.

Only 26 (10.6%) out of 246 sera showed antibody levels above 1000 ng/mL, reflecting a clinical specificity of 89.4%. Seropositivity was lowest within age decade 70 years and older (0%), followed by 2.5% (1/40) of decade 50–59 years, 4.8% (2/42) of decade 60–69 years, 5% (2/40) of decade 40–49 years, 12.5% (5/40) of decade 30–39 years, and 36.4% (15/44) of decade until 30 years (Table 3). Since sera of HPV L1 vaccinated women are identified by the serological test as well, all 26 positively tested sera of the control group were retested for the presence of HPV16 and HPV18 antibodies, assuming that the presence of antibodies against both subtypes would be an indicator for the presence of vaccine but not neoplasia-induced antibodies. Seventeen out of these 26 positively tested sera were tested positive for both, HPV16 and HPV18, antibodies. Nine out of these 26 sera were tested single positive, which means that these sera showed antibodies to HPV16 or HPV18 or other HPV high-risk subtypes (data not shown). Since to our knowledge, single positivity for only one HPV subtype has not been reported for vaccinees yet, these single positive controls were classified as “true positive” controls. These 17 sera were excluded from the specificity calculation reducing the number of control sera from 246 to 229. At the end, only 9 (3.9%) out of 229 sera were classified as true, nonvaccine associated, positives resulting in an overall specificity of 96.1% (range 92.1%–100%) at the 1000 ng/mL cutoff (Table 3).

Twenty-seven out of 229 sera showed antibody levels between 750 and 1000 ng/mL, which according to the instruction for use should be retested within 3–6 months to see if the antibody level shows an increase over time or not, as indicator of an active HPV-driven disease.

Nevertheless, to calculate the clinical specificity for the 750 ng/mL cutoff as well, we included these 27 “retesting positive” cases to the 26 positive cases of the 1000 ng/mL cutoff. Altogether, 53 (21.5%) out of 246 controls showed antibody levels above 750 ng/mL, reflecting a clinical specificity of 78.5%. Again, seropositivity was lowest within age decade 70 years and older (7.5%; 3/40), followed by 15.0% (6/40) of decade 50–59 years, 17.5% (7/40) of decade 30–39 years, 20% (8/40) of decade 40–49 years, 21.4% (9/40) of decade 60–69 years, and 45.5% (20/44) of decade until 30 years. Three additional out of these 27 sera were tested positive for both, HPV16 and HPV18, antibodies. Overall, 20 out of these 53 positively tested sera were classified as vaccine associated. These controls were excluded from the specificity calculation, reducing the number of control sera from 246 to 226. At the end, only 33 (14.6%) out of 226 sera were classified as true, nonvaccine associated, positives resulting in an overall specificity of 85.4% (range 78.6%–92.5%) at the 750 ng/mL cutoff (Table 4).

**Figure 1:**
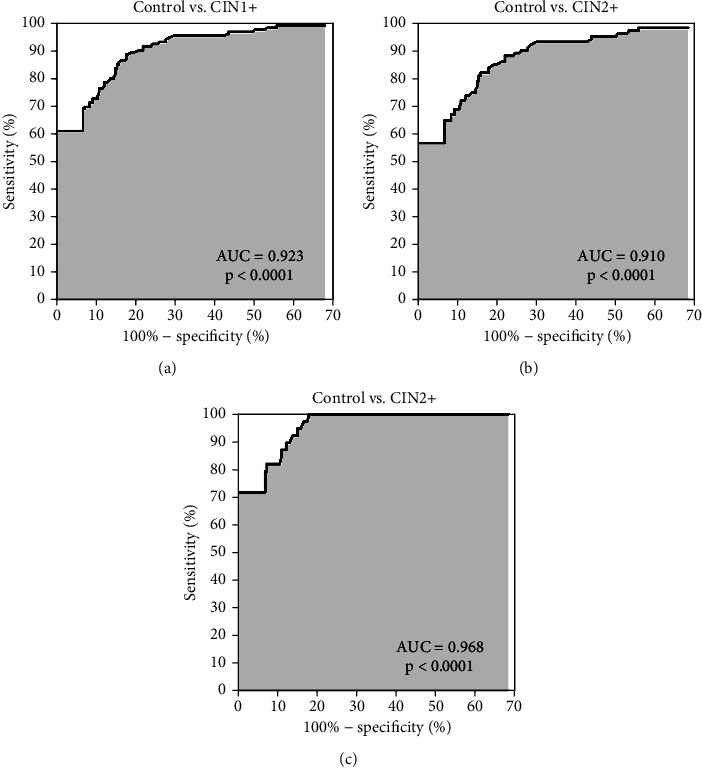
ROCs for evaluating the performance of determination of HPV L1 high-risk antibodies in serum of CIN1+ patients and controls. Comparison of (a) control and CIN1+, (b) control and CIN2+, and (c) control and CIN1/2; *p* < 0.0001.

The ROC analyses for different subsets of cases versus controls are shown in Figure 1. The AUC was calculated with 0.923 for all cases (CIN1+) versus all controls (*p* < 0.0001, Figure 1(a)), 0.910 for controls versus CIN2+ (*p* < 0.0001, Figure 1(b)), and with 0.968 for controls versus CIN1/2 (*p* < 0.0001, Figure 1(c)).

A correlation of the HPV high-risk antibody positive sera with the single positive HPV DNA subtype positive tissue samples confirmed the analytical specificity of the serological test for HPV 16, 18, 31, 33, 35, 39, 45, 51, 52, 53, 56, 58, 68, and 73.

## 4. Discussion

The WHO goal to eradicate HPV-induced diseases during the next century demands intensive efforts on several levels. Women especially from rural areas in LMICs suffer most from the disease, and it is common sense that high participation rates enabled by low-threshold access to vaccination, screening, and treatment are the three pillars for success. Nevertheless, how to reach women for primary screening purposes seems to be one of the biggest identified challenges, since the stated screening goal of 70% is significantly lower than the 90% goal for vaccination and treatment.

Primary HPV screening starting at the age of 30–35 years is established in many countries. This approach seems to be appropriate for low- or middle-income areas around the world as well, if the issues of the time-consuming logistic workflow from testing, reporting, and treatment can be solved by the local healthcare system, without losing the women at one or the other stage [15]. In this respect, easy access due to more comfortable sampling procedures lowering the barrier of participation, like urinary sampling, may have to be weighed against the gold standard of a vaginal smear, for example. Where it seems to be impossible to establish effective HPV DNA screening systems, robust, easy-to-use, and non–lab-associated alternative test systems like lateral flow rapid tests may be a complement to reach women that could not be reached otherwise.

Our case control study shows that an HPV high-risk lateral flow rapid test may have the potential to be used as a complement for the primary HPV DNA screening approaches. The serological assay is capable of detecting ≥ 89.9% of CIN1+, which is slightly superior compared to urinary-based HPV testing.

A specificity of 96.3% over all age groups (18–99 years) within a nonvaccinated female population suggests that the probability is very high that women with abnormal morphological findings are identified by the antibody response selectively. The AUC values of minimum 0.910 for controls versus CIN2+ emphasize this.

These high sensitivity and specificity findings could be explained by the viral life cycle [16]. The HPV L1 capsid protein is not produced during latent, morphologically asymptomatic HPV DNA positive infections, but selectively during early dysplastic lesions (CIN1 and 2).

During the active phase of the viral life cycle, the L1 capsid protein, together with L2, encapsidates the viral DNA after the replication has taken place. New infectious viral particles are assembled that are released at the superficial layer of the epithelium, serving as the antigen source for antibody production. Terminal differentiation of the basal epithelial host cell is essential for HPV L1 synthesis. Since this differentiation of the epithelial cell is lost during malignant progression of a precancerous lesion to cervical cancer, the L1 capsid protein is no longer produced in cervical cancer cases resulting in an inverse relationship regarding the severity of the lesion and L1 expression capability. This explains the lower sensitivity of the assay for more serious abnormalities as seen in CIN3 and cervical cancer cases compared to CIN1 and CIN2.

In line with the selective expression of the L1 capsid protein during the neoplastic development of cervical cancer, 3.9% of women are identified with a positive serological HPV L1 high-risk test result. This is in the middle of the range of 2.3%–5.9% cytological abnormalities (with and without ASCUS) that are typically found within screening populations [17]. Adjusting these 5.9% to the observed HPV high-risk frequency reported for such cases ends up at a positivity rate of 3.955%. This suggests that the probability is very high that women with abnormal morphological findings are selectively identified by the antibody response to CER818.

The discrimination between latent and neoplastic HPV infection leading to a high specificity is a major advantage of the serological approach since not every healthcare system in LMICs can handle a high follow-up transferal rate of about 25% when using HPV DNA screening approaches [15]. We have to keep in mind that HPV DNA test systems are not able to discriminate HPV DNA positive women with no morphological abnormalities or in other words “healthy women” from women with an HPV-induced neoplastic abnormality [18].

The COVID-19 pandemic has shown that lateral flow rapid tests are a good choice, if the benefit of receiving a test result within minutes is more important than a delayed reporting within the next few days.

In the setting of cervical cancer screening, an immediate test result offers the chance that a participating woman can be selectively identified, transferred to colposcopy, and treated in a “see and treat approach” within 1 h, if necessary. The “see and treat approach” therefore reduces the risk that the test result does no longer reach the women, which is especially important in rural areas where postal addresses are not self-evident.

In addition to the reported diagnostic metrics, lateral flow rapid tests offer several benefits that may be favorable for rural areas of LMICs as well. With a price below $5 per test, they are affordable, and no cooling chain is required, and a button cell battery enables the use of the reader for quantification of the test result in areas where electricity might be an issue.

A limitation of our initial case control study is the current setting, choosing a German study population of totally 405 cases and controls, which does not reflect the situation where the test is needed, in rural areas in LMICs that have not been screened and vaccinated yet. Therefore, larger cross-sectional studies are recommended to proof if the local German cutoff can be transferred one to one to other areas. This is especially of importance in areas where HIV coinfections may influence the antibody level due to low CD4 counts [19].

Altogether, the easy-to-use CER818-based serological HPV high-risk lateral flow rapid test may have the potential to assist in reaching the WHO screening goal of 70%. In addition, it seems to offer a chance to overcome the inequalities in access to screening tools that result in ethnic, racial, and social disparities in the incidence and mortality of cervical cancer.

## Figures and Tables

**Table 1:** Detailed characteristics of cases and controls.

**Patient characteristics**	**CIN1 ** **n** **(%)**	**CIN2 ** **n** **(%)**	**CIN3 ** **n** **(%)**	**CxCa ** **n** **(%)**	**Vaccinated ** **n** **(%)**	**Controls ** **n** **(%)**
*Age (years)*	Total 21	Total 18	Total 82	Total 17	Total 21	Total 246
Mean	44.7 (28–59)	37.6 (21–51)	39.7 (24–75)	50.2 (33–77)	27.8 (18–42)	45.8 (18–99)
18–29	1 (4.8)	1 (5.6)	6 (7.3)	0 (0)	14 (66.7)	44 (17.9)
30–39	5 (23.8)	8 (44.4)	43 (52.4)	4 (23.5)	6 (28.6)	40 (16.3)
40–49	8 (38.1)	8 (44.4)	21 (25.6)	6 (35.3)	1 (4.8)	40 (16.3)
50–59	7 (33.3)	1 (5.6)	11 (13.4)	2 (11.8)	0 (0)	40 (16.3)
60–69	0 (0)	0 (0)	0 (0)	3 (17.6)	0 (0)	42 (17.1)
70–79	0 (0)	0 (0)	1 (1.2)	2 (11.8)	0 (0)	40 (16.3)
*Pregnancies*	Total 21	Total 18	Total 82	Total 17	Total 21	n.a.
None	7 (33.3)	7 (38.9)	26 (31.7)	2 (11.8)	12 (57.1)	
Once	4 (19.0)	3 (16.7)	20 (24.4)	6 (35.3)	4 (19.0)	
≥ Twice	10 (47.6)	8 (44.4)	36 (43.9)	9 (52.9)	5 (23.8)	
*Children*	Total 21	Total 18	Total 82	Total 17	Total 21	n.a.
0	9 (42.9)	7 (38.9)	29 (35.4)	2 (11.8)	15 (71.4)	
1	3 (14.3)	6 (33.3)	22 (26.8)	8 (47.1)	2 (9.5)	
2	7 (33.3)	4 (22.2)	23 (28.0)	5 (29.4)	4 (19.0)	
≥ 3	2 (9.5)	1 (5.5)	8 (9.8)	2 (11.8)	0 (0)	
*Smoking*	Total 20	Total 17	Total 81	Total 17	Total 21	n.a.
No	12 (60.0)	11 (64.7)	53 (65.4)	8 (47.1)	13 (61.9)	
Yes	8 (40.0)	6 (35.3)	28 (34.6)	9 (52.9)	8 (38.1)	
*Contraceptives (pill)*	Total 20	Total 18	Total 79	Total 15	Total 21	n.a.
No	11 (55.0)	6 (33.3)	44 (55.7)	13 (86.7)	9 (42.9)	
Yes	9 (45.0)	12 (66.7)	35 (44.3)	2 (13.3)	12 (57.1)	

**Table 2:** Seropositivity of cases, vaccinees, and controls at cutoff 750 and 1000 ng/mL CER818.

**Positive test results at**		**Cutoff**	
**Diagnosis**	**Total**	**750 ng/mL**	**1000 ng/mL**
CIN1	21	21 (100%)	18 (85.7%)
CIN2	18	18 (100%)	14 (77.8%)
CIN3	82	71 (86.6%)	55 (67.1%)
CxCa	17	14 (82.4%)	13 (76.5%)
Total	138	124 (89.9%)	100 (72.5%)
Vaccines	21	21 (100%)	20 (95.2%)
Controls	Total	750 ng/mL	1000 ng/mL
< 30 years	44	4 (9.1%)	16 (36.4%)
30–39 years	40	2 (5.0%)	5 (12.5%)
40–49 years	40	6 (15.0%)	2 (5.0%)
50–59 years	40	5 (12.5%)	1 (2.5%)
60–69 years	42	7 (16.7%)	2 (4.8%)
70+ years	40	3 (7.5%)	0 (0%)
Total	246	27 (11.0%)	26 (10.6%)

**Table 3:** Specificity calculation of the seropositive controls at cutoff 1000 ng/mL, by identification of HPV16/18 double versus HPV16/18/HR HPV other single positives as indicator for vaccine versus neoplasia-induced antibodies classified as “true positives”.

**Positive test results at**		**Cutoff**	**HPV16/HPV18 positivity**	**HPV16/18/HR HPV other positivity**	
**Controls**	**Total**	**S** **e** **r** **o** **p** **o** **s** **i** **t** **i** **v** **i** **t** **y** > 1000 **ng/mL**	**Vaccinees (double positive)**	**True positives (single positive)**	**Specificity for true positives**
< 30 years	44	16 (36.4%)	15/44 (34.1%)	1/29 (3.4%)	96.6%
30–39 years	40	5 (12.5%)	2/40 (5.0%)	3/38 (7.9%)	92.1%
40–49 years	40	2 (5.0%)	0/40 (0%)	2/40 (5.0%)	95.0%
50–59 years	40	1 (2.5%)	0/40 (0%)	1/40 (2.5%)	97.5%
60–69 years	42	2 (4.8%)	0/42 (0%)	2/42 (4.8%)	95.0%
70+ years	40	0 (0%)	0/40 (0%)	0/40 (0%)	100%
Total	246	26 (10.6%)	17/246 (6.9%)	9/229 (3.9%)	96.1%

**Table 4:** Specificity calculation of the seropositive controls at cutoff 750 ng/mL, by identification of HPV16/18 double versus HPV16/18/HR HPV other single positives as indicator for vaccine versus neoplasia-induced antibodies classified as “true positives”.

**Positive test results at**		**Cutoff**	**HPV16 + HPV18 positivity**	**HPV16/18/HR HPV other positivity**	
**Controls**	**Total**	**S** **e** **r** **o** **p** **o** **s** **i** **t** **i** **v** **i** **t** **y** > 750 **ng/mL**	**Vaccinees (double positive)**	**True positives (single positive)**	**Specificity for true positives**
< 30 years	44	20 (45.5%)	17/44 (38.6%)	3/27 (11.1%)	88.9%
30–39 years	40	7 (17.5%)	2/40 (5%)	5/38 (13.2%)	86.8%
40–49 years	40	8 (20.0%)	0/40 (0%)	8/40 (20.0%)	80.0%
50–59 years	40	6 (15.0%)	1/40 (2.5%)	5/39 (12.8%)	87.2%
60–69 years	42	9 (21.4%)	0/42 (0%)	9/42 (21.4%)	78.6%
70+ years	40	3 (7.5%)	0/40 (0%)	3/40 (7.5%)	92.5%
Total	246	53 (21.5%)	20/246 (8.1%)	33/226 (13.3%)	86.7%

## Data Availability

Raw experimental data associated with the figures presented in the manuscript are available from the corresponding author upon reasonable request.
